# Contribution of *P*. *falciparum* parasites with *Pfhrp* 2 gene deletions to false negative PfHRP 2 based malaria RDT results in Ghana: A nationwide study of symptomatic malaria patients

**DOI:** 10.1371/journal.pone.0238749

**Published:** 2020-09-04

**Authors:** Linda Eva Amoah, Benjamin Abuaku, Abagna Hamza Bukari, Donu Dickson, Eunice Obeng Amoako, George Asumah, Alexander Asamoah, Nana Yaw Preprah, Keziah Laurencia Malm

**Affiliations:** 1 Dept. of Immunology, Noguchi Memorial Institute of Medical Research, University of Ghana, Accra, Ghana; 2 Dept. of Epidemiology, Noguchi Memorial Institute of Medical Research, University of Ghana, Accra, Ghana; 3 National Malaria Control Program, Accra, Ghana; Instituto Rene Rachou, BRAZIL

## Abstract

**Introduction:**

False-negative malaria rapid diagnostic test (RDT) results amongst symptomatic malaria patients are detrimental as they could lead to ineffective malaria case management. This study determined the nationwide contribution of parasites with *Pfhrp2 and Pfhrp 3 gene* deletions to false negative malaria RDT results in Ghana.

**Methods:**

This was a cross sectional study where whole blood (~2 ml) was collected from patients presenting with malaria symptoms at 100 health facilities in all the regions in Ghana from May to August 2018. An aliquot of the blood was used to prepare thin and thick blood smears, filter paper blood spots (DBS) and spot a PfHRP 2 RDT kit. The remaining blood was separated into plasma and blood cells and stored at -20°C. *Plasmodium* parasite density and species identity was estimated from the blood smears. *Plasmodium falciparum* specific *18S rRNA* PCR, *merozoite surface protein* (*msp* 1) and *glutamate rich protein* (*glurp*) gene PCR were used to identify *P*. *falciparum* positive samples, which were subjected to *Pfhrp 2*/3 exon1-2 and exon2 genotyping.

**Results:**

Of the 2,860 microscopically *P*. *falciparum* positive patients analyzed, 134 (4.69%) had false negative *P*. *falciparum* specific RDT results. Samples for PCR analysis was available for 127 of the false negative patients, and the analysis identified 116 (91.3%) as positive for *P*. *falciparum*. Only 58.1% (79/116) of the false negative RDT samples tested positive by *msp* 1 and *glurp* PCR. Genotyping of exon 1–2 and exon 2 of the *Pfhrp* 2 gene identified 12.9% (10/79) and 39.5% (31/79) of samples respectively to have deletions. Genotyping exon 1–2 and exon 2 of the *Pfhrp* 3 gene identified 15.2% (12/79) and 40.5% (32/79) of samples respectively to have deletions. Only 5% (4/79) of the false negative samples had deletions in both exon 1–2 and exon 2 of the *Pfhrp 2 gene*. Out of the 49 samples that tested positive for aldolase by luminex, 32.6% (16/49) and) had deletions in *Pfhrp* 2 exon 2 and 2% (1/49) had deletions in both exon 2 and exon 1–2 of the *Pfhrp* 2 gene.

**Conclusions:**

The low prevalence of false negative RDT test results provides assurance that PfHRP 2 based malaria RDT kits remain effective in diagnosing symptomatic malaria patients across all the Regions of Ghana. Although there was a low prevalence of parasites with deletions in exon 2 and exon 1–2 of the *Pfhrp* 2 gene the prevalence of parasites with deletions in *Pfhrp* 2 exon 2 was about a third of the false negative RDT results. The need to ensure rapid, accurate and reliable malaria diagnosis requires continuous surveillance of parasites with *Pfhrp* 2 gene deletions.

## Introduction

The diagnosis of symptomatic malaria is typically based on smear microscopy, the gold standard [1]. However, alternative diagnostic tests such as the rapid diagnostic test (RDT) have been developed for malaria diagnosis, especially in low-income endemic countries where skilled microscopists and resources to perform microscopy are limited [2, 3]. In 2010, Ghana implemented the World Health Organisation (WHO) recommendation to confirm all suspected malaria cases by either microscopy or RDT prior to administering antimalarial drugs [4, 5]. The most commonly used malaria RDTs detect histidine-rich protein 2 (PfHRP 2) antigen, which is specific for the diagnosis of *Plasmodium falciparum* parasites [6], the causative agent for over 90% of all malaria cases in sub-Saharan Africa [7].

Despite the numerous advantages of malaria RDTs over smear microscopy, a few disadvantages exist. Some disadvantages include prolonged PfHRP2 antigen persistence after parasite clearance, which results in false positive test results as well as the prozone effect [8] and the presence of parasites with deletions in the *Pfhrp* 2 gene, which codes for the PfHRP 2 antigen, which both cause false negative RDT test results [9, 10]. Parasites with deletions in the *Pfhrp 2* gene have been found to react positively with PfHRP 2-based malaria RDTs due to cross-reactivity of PfHRP 2 with PfHRP 3 [11]. The PfHRP 3 protein is a structural homologue of PfHRP 2, with almost 95% sequence identity to *Pfhrp 2* [12].

Deletions in the *Pfhrp 2* gene that result in false negative malaria RDT results are detrimental and have the potential of missing malaria positive patients in countries like Ghana where testing is largely by PfHRP2 RDTs. The symptomatic malaria patients who test negative may likely not receive appropriate antimalarial treatment [13, 14]. To ensure the maintenance of the diagnostic accuracy of malaria RDT results and prevent the misdiagnosis of malaria in the era of the WHO’s Test Treat and Track initiative, the WHO partnered with the Foundation for Innovative New Diagnostics (FIND) to set up the WHO-FIND Malaria Evaluation Programme to provide quality assurance for the manufacture of malaria RDT kits [15].

Since deletions in the *Pfhrp* 2 gene can adversely affect the diagnostic accuracy of PfHRP 2 based malaria RDT kits, the WHO has recommended that countries with a *Pfhrp* 2 gene deletion parasite population at or above 5% should resort to the use of non-PfHRP 2-based RDT kits [16]. Identifying parasites with *Pfhrp* 2 gene deletions in symptomatic malaria infections from high endemic countries such as Ghana is complicated by the fact that the infections are predominantly multiclonal [17] and the presence of a minor population of parasites with *Pfhrp* 2 gene deletions can be masked by the presence of co-infecting parasites with intact *Pfhrp* 2 genes.

The first reports of the existence of parasites with deletions in the *Pfhrp 2* gene was in the Amazon region of Peru [9] and has presently been reported in numerous malaria endemic countries [16], including Ghana [18]. To date there have been very few reports of the existence of parasites with deletions in *Pfhrp 2* in Ghana, most of which have been conducted on asymptomatic malaria carriers and also without implementing the WHO recommendations [18, 19]. Due to the implications of the presence of such parasites amongst symptomatic malaria patients, a nationwide survey was conducted on suspected malaria patients attending 100 health facilities across the country to identify the presence and distribution of parasites with deletions in the *Pfhrp* 2 and or *Pfhrp 3* gene that result in false negative PfHRP 2-based malaria RDT results.

## Methods

### Ethics

Ethical approval for this study was obtained from the Institutional Review Board of the Noguchi Memorial Institute for Medical Research, University of Ghana (study # 068/17-18). Written informed consent was obtained from each adult participant (≥18years old) and caregivers of participants less than 18 years old provided parental consent. Written informed assent was also obtained from all participants aged between 12 and 17 years old. Each consenting or assenting participant was informed of the objectives, methods, anticipated benefits and risks of the study as well as their liberty to withdraw without any penalty.

### Study design

This was a cross-sectional survey among all symptomatic individuals seeking care in selected health care facilities in the ten regions of Ghana between May and August 2018.

### Study site and population

**Fig 1:**
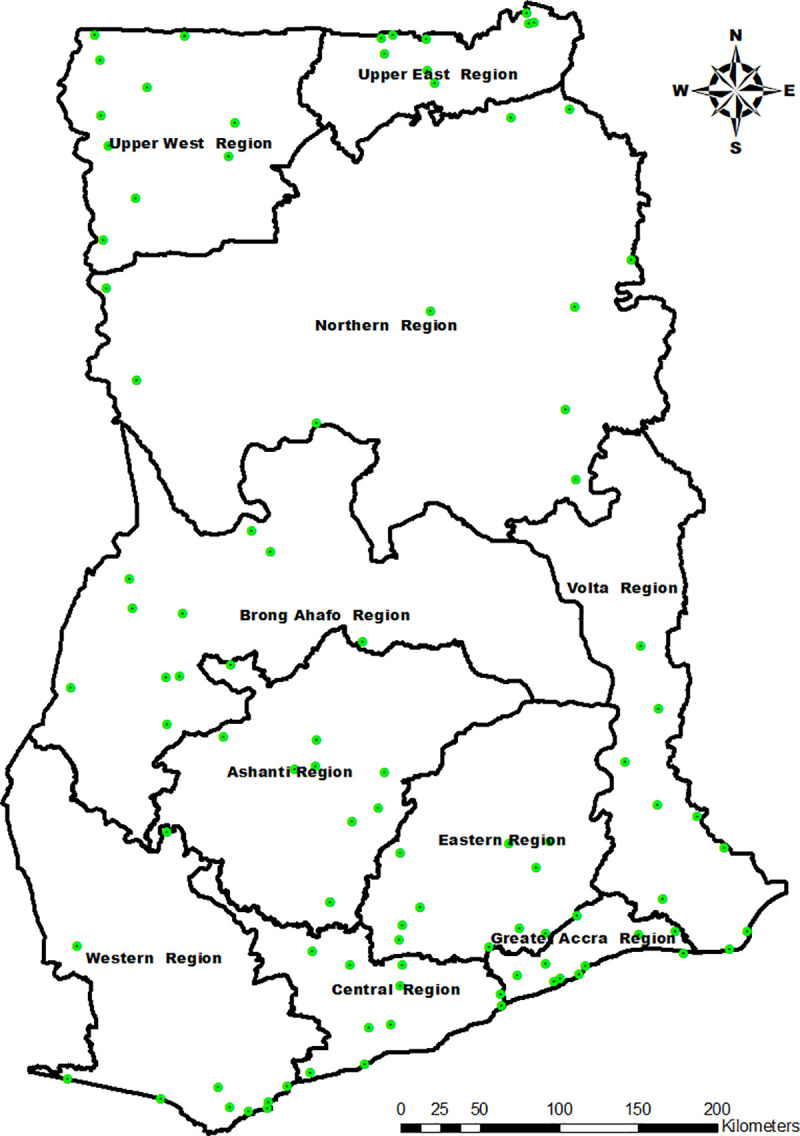
Map of Ghana highlighting the study sites. Study sites are represented on the map by light green circles with a black star in each of them. The map was created by Mr George Asumah Adu, NMCP. Shapefiles for the map was obtained from the Centre for Sensoring and Geographc information Services (CERGIS). ArcGIS 10.3.1 was used to plot the GPS coordinates.

As per the WHO protocol on determining the prevalence of *Pfhrp 2*/3 gene deletions [20], a total of 10 health care facilities were randomly selected in each of the 10 Regions of Ghana (Fig 1). The selection was based on probability proportional to size estimates (PPSE) using average monthly outpatient department (OPD) suspected malaria cases for 2016 to represent facility size (data source: DHIMS 2). The sampling was designed to yield a selection of 3 district/municipal hospitals and 7 health centres in each of the 10 Regions. Each patient suspected to have uncomplicated malaria who presented to the selected health facilities during the study period and gave his/her consent was included in the study.

### Sample collection and processing

After obtaining written informed consent (for adult participants), parental consent (for children under 18 years old), and assent (for children 12 to 17 years old) the study participants were enrolled into the study. Demographic data was obtained from each participant and entered in KoBoCollect and about 2 ml of venous blood collected into an EDTA vacutainer**®** tube. The venous blood was tested on the CareStart**®** malaria PfHRP 2 RDT kit (AssessBio, CA, USA). Samples were classified as RDT positive if both the control and test lines were positive and RDT negative if only the control line but not the test yielded a positive result.

Thick and thin blood smears were also prepared using 6 μl and 2 μl of whole blood respectively. Filter paper blood spots were prepared by spotting four 50 μl drops of whole blood on to Whatman**®** No. 3 filter paper. The blood spots were air dried and stored individually in a Ziplock**®** bag with a desiccant. Packed cells were isolated from the remaining blood samples and stored in a 1.5 ml eppendorf**®** tube at -20°C until used.

### Estimation of parasite density

The blood smears were processed and then stained with 3% Giemsa for 45 min. The stained slides were subsequently viewed under 100X oil immersion microscope [21]. Two independent (WHO level 1 and 2 certified) microscopists read the slides. Slides discordant for parasite and or species identification were re-examined by a third expert microscopist. Two examinations with the same results were considered as final. Parasite quantification was based on counting parasites against 200 white blood cell counts (WBCs) when ≥ 100 parasites were observed or against 500 WBCs when <100 parasites were observed per thick film assuming 8,000 WBCs were contained in 1 μl of blood. Else when ≥ 100 parasites were observed in each thick film field, parasite quantification was based on the number of parasitized red blood cells (RBCs) counted against 5000 total RBCs using the thin film. This was then computed per μl of blood assuming 5,000,000 RBCs/μl of blood [22].

### DNA extraction

Samples that were negative by the routinely used CareStart PfHRP 2 RDT^**®**^ and positive by microscopy (false negative RDT samples) were sorted out from the total samples collected. Genomic DNA was extracted from the dry blood spots (DBS) using the Chelex extraction method [23] and from whole blood using the gDNA mini kit (Zymo Research, USA) according to manufacturer’s instructions. Briefly, 100 μl of whole blood was mixed with 400 μl of genomic lysis buffer supplemented with 0.05% β mercaptoethanol in a 1.5 ml tube. The DNA was eluted using 50 μl of DNA elution buffer. The eluted DNA was stored at -20°C until use.

### Nested PCR detection of *Plasmodium falciparum*

The nested PCR amplification of the *18S rRNA* gene was adapted from Singh *et al*. [24] with slight modification [25]. Briefly, 200 nM dNTPs, 2.5 mM MgCl_2_, 133 nM each of forward (rPLU6) and reverse (rPLU5) primers (S1 Table) and 1 U OneTaq DNA polymerase (New England Biolabs, UK) was used to amplify the *18S rRNA* gene from 2 μl of DNA obtained using the Zymo extraction kit or 5 μl of Chelex extracted DNA (~40 ng) in the primary reaction. The nested PCR was performed using similar concentrations of reagents as in the primary reaction mix; however, rFal1 (forward) and rFal2 (reverse) primers were used to amplify 0.5 μl of the primary product in *P*. *falciparum* identification. The cycling parameters for the primary and nested reactions are listed in S1 Table. Genomic DNA from the 3D7 strain of *P*. *falciparum* (MRA 102G) was used as a positive control and the no template control served as negative control.

### Parasite genotyping

#### *Msp* 1 and *glurp* loci

The WHO malaria parasite genotyping protocol [26] was followed with slight modifications as described by Ayanful-Torgby *et al*. [27]. Briefly, amplifications were carried out in 15 μl volumes for both the primary and nested reactions. The *msp* 1 locus was amplified using nested PCR. The primary reaction contained 200 nM each of M1-0F and M1-0R primers in a reaction containing 2 μl of DNA obtained using the Zymo extraction kit or 5 μl of Chelex extracted DNA (~40 ng) and 0.1 μl One Taq DNA polymerase (NEB, Hertfordshire, UK). In the secondary reaction, 1 μl of the primary PCR product was amplified using 200 nM each of M1-KF+M1-KR (K1 alleles), M1- R033R+M1-R033F (R033 alleles) and M1-MF+M1-MR (Mad20 alleles) primer sets (S1 Table).

The *glurp* gene was amplified using semi nested PCR. In the primary reaction, 500 nM each of primers G-F3 and G-F4 was used to amplify 3 μl (20–30 ng) of gDNA. Primers G-F4 and GNF (500 nM each) were used in the secondary reaction of amplify 2 μl of the primary PCR product. The cycling conditions for the primary and secondary PCR reactions included an initial denaturation at 95°C for 5 min followed by 35 cycles of a denaturation at 94°C for 30 seconds, primer annealing at 55°C (for primary) or 58°C (for secondary) for 1 minute and an extension at 68°C for 1 minute and a final extension at 68°C for 5 minutes.

#### *Pfhrp 2* and *Pfhrp 3* loci

Exons 2 and 1–2 of the *Pfhrp 2* and *Pfhrp 3* genes were amplified using the protocol described by Abdallah *et al*.,[28] with minor modifications. Briefly for the exon 2 amplifications, 0.5 μM of primers *Pfhrp2*F1 (forward) and *Pfhrp2*R1 (reverse) primers for *Pfhrp2* exon 2 and *Pfhrp3*F1 (forward) and *Pfhrp3*R1 (reverse) primers for *Pfhrp 3* exon 2 were used to amplify 2 μl of DNA obtained using the Zymo extraction kit or 5 μl of Chelex extracted DNA (~40 ng) using 0.1 μl OneTaq DNA polymerase (NEB, Hertfordshire, UK) in a 15 μl reaction containing 1.8 mM MgCl_2_ and 0.2 μM dNTP mix. The DNA was initially denatured at 95#x00B0;C for 3 min followed by 35 cycles of denaturation at 94°C for 30 seconds, annealing at 58°C (*Pfhrp 2* gene) or 55°C (*Pfhrp 3* gene) for 30 seconds and an extension at 68°C for 1 min. The final extension was performed at 68°C for 5 min and finally held at 4°C. Genomic DNA from Dd2 (CDC, USA), HB3 (CDC, USA) and 3D7 (CDC, USA), representing *Pfhrp 2* deleted, *Pfhrp 3* deleted and wild type parasites respectively were used as controls for the PCR amplifications.

A nested PCR protocol was used to amplify exon 1–2 of the *Pfhrp 2* and *Pfhrp 3* genes. The primary PCR reactions were carried out in a 15 μl reaction volume containing 2 μl of DNA obtained using the Zymo extraction kit or 5 μl of Chelex extracted DNA (~40 ng), 0.2 μM each of forward and reverse primers 2E12F1 and 2E12R1 for *Pfhrp 2* and 3E12F1 and 3E12R1 for *Pfhrp 3* respectively), 0.133 μM dNTPmix and 2.5 mM MgCl_2_ supplemented with 0.1 μl of OneTaq DNA polymerase (NEB, Hertfordshire, UK). The secondary reactions were amplified using similar reaction conditions as the primary reaction, however, the primer sets were replaced with 2E12F2 and 2E12R2 for *Pfhrp 2* or 3E12F2 and 3E12R2 for *Pfhrp 3*. The templates for the secondary reactions were 2 μl of a 1:100 dilution of the respective primary PCR products.

#### Resolution of PCR amplicons

PCR products (10 μl) were separated on 2.0% agarose gels containing 0.2 μg/ml ethidium bromide and subsequently viewed under UV illumination using a Bio-Print TX4 gel documentation system (Vilber, Wielandstrasse, Germany). All samples that yielded visible fragments after agarose gel electrophoresis were classified as positive for the particular PCR reaction.

#### Estimation of Aldolase and PfHRP 2 antigen levels

The Multiplex Bead Assay (MBA) was used to determine the PfHRP 2 and pan-Aldolase levels of the samples [29]. Briefly, one 6 mm disc was punched out of the DBS and incubated overnight in 200 μl of elution buffer (0.05% phosphate-buffered saline (PBS), Tween 20, 0.5% Bovine serum albumin, 0.02% sodium azide (NaN_3_), 50 ml liquid casein, 5 g polyvinyl alcohol, 5 g polyvinylpryrrolidine). Subsequently, 50 μl of PfHRP 2 and pan-Aldolase coated beads (1:500 dilution of each in elution buffer) was aliquoted into each well of the plate. The samples tested included a 50 μl aliquot of the test samples and negative control samples from 86 malaria naïve people. The plates were washed three times with wash buffer. Anti-PfHRP 2 and anti-Aldolase (1:500) detection antibodies (50 μl) were then added to each well of the plate and Incubated for 45 mins with shaking. The plate was subsequently washed three times and then incubated for 30 mins with 50 μl of streptavidin-phycoerythrin (1:200) with shaking. After the final wash, the plates were read on the Luminex 200 (Luminex Corp. USA). The cutoff value for PfHRP 2 and pan-Aldolase antigen positivity was the lognormal mean fluorescence intensity (MFI) of the 86 malaria naïve samples minus the background (bg) MFI signal ± 3 SD.

### Data analysis

The datasets were captured using KoBoCollect, extracted and cleaned in IBM SPSS version 22. IBM SPSS version 22 was used to generate the descriptive statistics including mean, standard error of the mean and the frequency. Kruskal-Wallis test (GraphPad Prism v5) was used to compare the median Regional parasite densities and the age of the study participants. Pearson’s Chi squared test was used to compare the medians of the Regional distribution of male and female participants at a 5% level of significance.

The MFI cut off value for positivity for the PfHRP2 and aldolase luminex data was set at 50 and 20 respectively. Spearman’s correlation (GraphPad Prism v5) was used to determine the correlation between parasite density and parasite antigen (PfHRP 2 and aldolase) concentrations.

## Results

**Fig 2:**
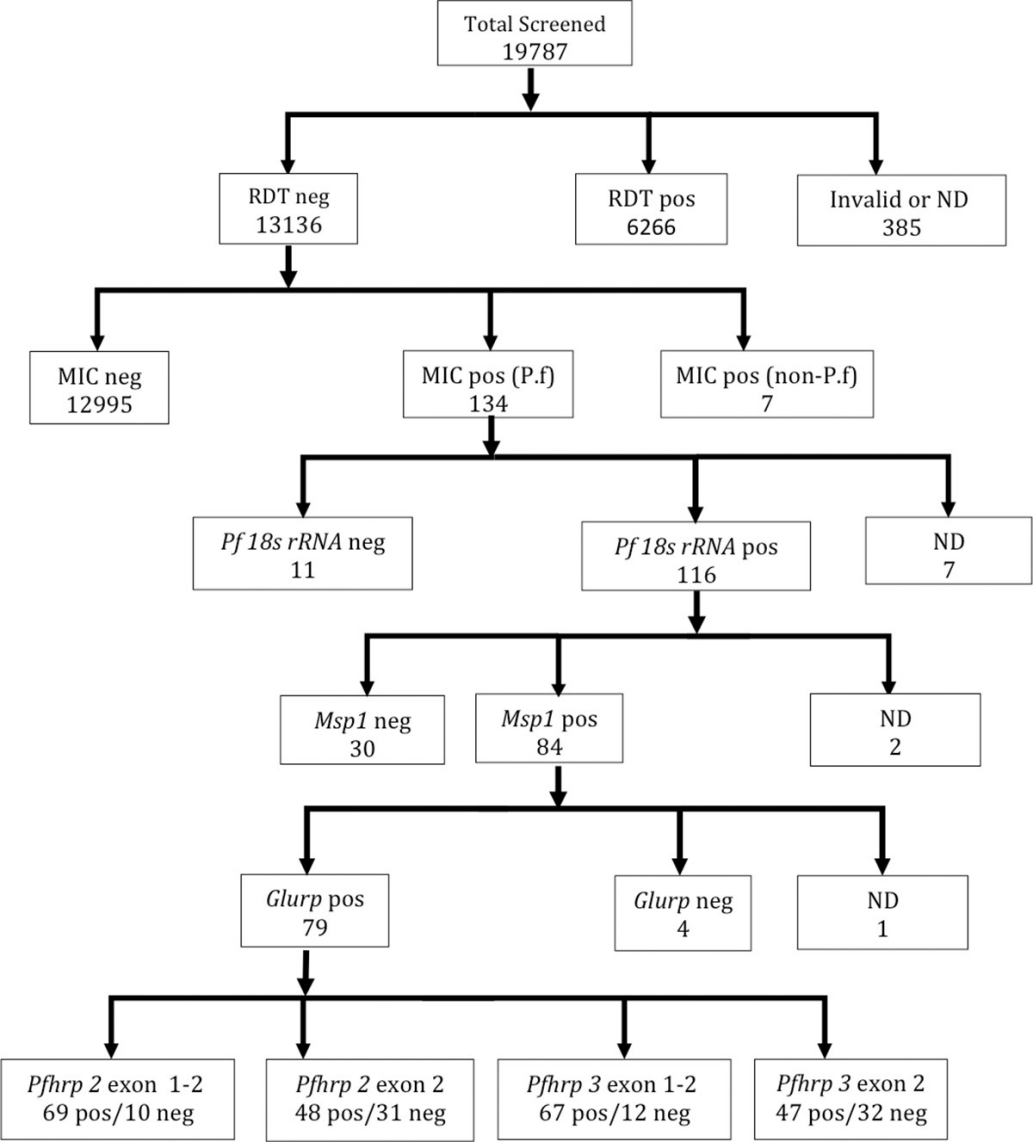
A flow chart illustrating sample processing. A schematic of the processes used to identify the false negative and subsequently the *Pfhrp* 2 and *Pfhrp 3* gene deletion samples as well as the number of samples processed at each step. RDT, PfHRP 2 based malaria rapid diagnostic test kit; MIC, microscopy; pos, positive; neg, negative; ND, not determined.

Microscopy identified the presence of malaria parasites in 2890 of the 18618 samples with valid microscopy results. Microscopic densities of *Plasmodium* parasites were identified in 1% (141/13136) of the samples that tested negative by PfHRP 2 RDT kits (Fig 2). Out of these 141, seven samples were non-*P*. *falciparum* infections (by microscopy), leaving 134 false negative (FN) samples. Unfortunately, neither DBS nor whole blood was found for seven of these samples. The age of the patients with FN results ranged between 1 and 94 years and the age distribution were similar across the 10 Regions (Kruskal-Wallis test, p = 0.3). Children aged ≤10 years constituted 22.6% and adults 50 years and above constituted 15.8% of the study population. The distribution of males and females across the Regions were similar (Chi Square = 7.372, p = 0.497), with males representing about 30% of the study population.

The distribution of people carrying parasites that were identified as FN was nationwide, with the highest prevalence found in the Ashanti Region and the lowest prevalence in the Volta Region (Table 1). The parasite densities of the FN samples ranged from 32 to 641,600 p/μl and were significantly different across the 10 Regions, Kruskal-Wallis test, p = 0.048). There were 12 samples with parasite densities below 200 p/μl.

**Table 1:** Demographic characteristics of the 127 study participant with false negative RDT results by Region.

	A (23)	BA (8)	C (26)	E (6)	GA (6)	N (17)	UE (12)	UW (19)	V (3)	W (7)	Total (127)
Sex											
Male	4	2	8	3	4	5	2	9	0	2	39
Female	19	6	18	3	2	12	5	10	3	4	82
AGE (yrs)											
Mean	27.2	31.8	32.5	45.2	19.0	24.9	17.7	32.5	28.5	12.5	28.4
SEM	4.7	8.1	5.1	13.3	4.2	3.7	6	6.2	7.7	6.4	2.1
Minimum	1	3	2	4	4	2	1	2	10	2	1
Maximum	84	76	85	84	33	62	48	94	43	43	94
PD (p/μl)											
Mean	44346.7	25209.4	7567.6	64231.2	3510.7	99341.2	82547.0	128846.4	61323.3	62353.0	59328.6
SEM	13656.4	19050.3	4079.7	48969.2	1832.7	44525.8	40934.6	39788.2	46221.6	28398.1	10374.9
Minimum	76	357	32	1842	360	95	32	692	7803	144	32
Maximum	249320	157775	106800	307500	12431	580000	452500	641600	199612	190000	641600

SEM, standard error of the mean; Min, minimum; Max, maximum; M, male; F, female; PD, parasite density. A, Ashanti; BA, Brong Ahafo; C, Central; E, Eastern; GA, Greater Accra; N, Northern; UE, Upper East; UW, Upper West; V, Volta; W, Western. Demographic data was unavailable for 5 people from the Upper East and 1 person from the Western Region.

### Molecular characterization

Whole blood or filter paper samples were available for 127 out of the 134 samples, unfortunately neither whole blood nor DBS could be found for 7 samples.

*Plasmodium falciparum* species PCR identified 116 out of the 127 FN samples as positive (S2 Table). Further PCR analysis of these 116 samples identified 85 and 102 samples that tested positive for *msp* 1 and *glurp* genes respectively. However, only 79 samples tested positive by both *msp* 1 and *glurp* (Fig 2). The 79 samples included 7 samples that contained parasite densities of less than 200 p/μl.

Genotyping of both *Pfhrp* 2 and *Pfhrp* 3 genes at exon 2 and exon1-2 were performed on the 79 samples that tested positive for both *glurp* and *msp* 1. Amplification of exon 2 of both *Pfhrp* 2 and *Pfhrp* 3 yielded fewer positive samples than the amplification of exon1-2 (Table 2). There were 31 samples overall that did not yield amplicons after *Pfhrp* 2 exon 2 PCR (S2 Table). The only Region where no sample was identified to have a deletion in exon 2 of the *Pfhrp* 2 gene was the Eastern Region (Table 2).

**Table 2:** Regional distribution of *Pfhrp* 2/3 negative samples.

	A	BA	C	E	GA	N	UE	UW	V	W	TOTAL
Hrp2 1–2	3	1	4	0	0	0	2	0	0	0	10
Hrp3 1–2	2	1	5	0	1	1	1	0	0	1	12
Hrp3 E2	4	5	9	1	1	5	2	3	1	1	32
Hrp2 E2	9	3	5	0	2	5	1	4	1	1	31
Hrp2 E2/E1-2	1	1	2	0	0	0	0	0	0	0	4
Hrp2/3 E2	2	2	4	0	1	1	0	2	1	1	14

Hrp2, *Pfhrp* 2; Hrp3, *Pfhrp* 3; E, exon; A, Ashanti; BA, Brong Ahafo; C, Central; E, Eastern; GA, Greater Accra; N, Northern; UE, Upper East; UW, Upper West; V, Volta; W, Western.

The numbers represent the number of samples that failed to yield amplicons after the respective PCR reactions.

Seven (8.9%) of the 79 false negative samples contained infections with parasite densities of less than 200 p/μl (between 76 and 192 p/μl). All these 7 samples tested negative after *Pfhrp 3* exon 2 PCR, whilst 3 samples tested negative for *Pfhrp* 2 exon 2 (Table 3). The samples with low parasite density (<200 p/μl) were from four (Ashanti, Central, Northern and the Upper East) of the 10 regions.

**Table 3:** Distribution of parasites with *Pfhrp* 2/3 gene deletions in samples containing less than 200 parasites/μl.

		*Pfhrp* 2		*Pfhrp* 3		LUMINEX		PD
ID	Region	E2	E1-2	E2	E1-2	PfHRP 2	Aldo	p/μl
A	A	0	1	0	1	-	-	76
B	A	1	0	0	1	-	+	89
C	N	1		0	1	+	-	95
D	C	0	0	0	0	-	-	96
E	C	1	1	0	1	-	-	128
F	C	0	1	0	0	-	-	176
G	UE	1	0	0	1	ND	ND	192

A, Ashanti; C, Central; UE, Upper East. The prevalence of *Pfhrp* 2/3 gene deletions amongst samples with parasite densities <200 p/μl. The numbers represent exact counts of samples that tested negative for each of the PCR reactions. ND, not determined.

Out of the 31 samples identified with *Pfhrp* 2 exon 2 gene deletions, only 4 (12.9%) tested negative for *Pfhrp* 2 exon1-2. Samples with gene deletions in both *Pfhrp* 2 exon 2 and *Pfhrp* 2 exon 1–2 were found in only three Regions, the Ashanti, Brong Ahafo and the Central, with the Central Region having the highest prevalence (50%, 2/4) of samples with deletions in both *Pfhrp* 2 exon 2 and exon 1–2 (Table 2).

Almost half (14/31) of the samples with *Pfhrp* 2 exon 2 gene deletions also had deletions in *Pfhrp* 3 exon 2. All the parasites with *Pfhrp* 2 exon 2 gene deletions in the Upper East, Volta and Western also had deletions in *Pfhrp* 3 exon 2.

#### Serological characterization

The PfHRP 2 antigen was absent (MFI values less than 50) in 32.9% (26/79) of the FN samples, whilst the aldolase antigen was absent (MFI values less than 20) in 38% (30/79) of the FN samples. There were 27.8% (22/79) of the FN samples that were negative for both PfHRP 2 antigen and aldolase.

About 68% (48/71) of the samples that had parasite densities >200 p/μl tested positive by aldolase luminex. Parasite density exhibited a significant strong positive correlation (Spearman’s R = 0.715, p = 0.0001) with aldolase concentrations and a significant but weak correlation (Pearson R = 0.279, p = 0.008) with PfHRP 2 antigen concentration.

The distribution of parasites with *Pfhrp* 2/3 gene deletions amongst samples that tested positive for aldolase by luminex were predominant in the Ashanti Region and Northern Region (Tables 4 and 5).

**Table 4:** Serological assessment of the samples.

	A	BA	C	E	GA	N	UE	UW	V	W	TOTAL
PfHRP 2e2 neg	5	5	9	0	1	3	1	2	0	0	26
PfHRP 2e2 pos	10	1	2	2	2	10	8	11	3	4	53
PD >200/*Pfhrp* 2e2 neg	3	5	6	0	1	3	0	2	0	0	20
Aldo neg	5	4	10	0	2	4	1	3	0	1	30
Aldo neg/ *Pfhrp* 2e2 pos	2	0	2	0	1	1	0	1	0	1	8
Aldo neg/ *Pfhrp* 2e2 neg	3	4	8	0	1	3	1	2	0	0	22
Aldo pos	10	2	1	2	1	9	8	10	3	3	49
Aldo pos/ *Pfhrp* 2e2 pos	4	2	1	1	2	5	7	8	2	3	33
Aldo pos/ *Pfhrp* 2e2 neg	6	0	0	1	2	4	1	2	1	1	16
PD >200/Aldo pos	9	2	1	2	1	9	8	10	3	3	48

HRP, *Pf*HRP *2 antigen;* Aldo, aldolase antigen; neg, negative; pos, positive; PD, parasite density; A, Ashanti; BA, Brong Ahafo; C, Central; E, Eastern; GA, Greater Accra; N, Northern; UE, Upper East; UW, Upper West; V, Volta; W, Western. The numbers represent frequency as exact counts.

**Table 5:** Regional distribution of *Pfhrp* 2/3 negative parasites in aldolase positive samples.

			Luminex		*Pfhrp* 2		*Pfhrp 3*	
ID	Region	PD (p/μl)	PfHRP 2	Aldo	exon 2	exon 1–2	exon 2	exon 1–2
*Pfhrp* 2 exon 2 negative								
1	A	3200	0	1	0	1	0	1
2	A	22825	1	1	0	0	1	1
3	A	29099	1	1	0	1	1	1
4	A	25004	1	1	0	1	0	1
5	A	47407	1	1	0	1	1	0
6	A	30857	1	1	0	1	0	1
7	GA	1686	1	1	0	1	0	1
8	GA	12431	1	1	0	1	1	1
9	N	986	1	1	0	1	1	1
10	N	57490	1	1	0	1	1	1
11	N	32941	1	1	0	1	1	1
12	N	41760	1	1	0	1	1	1
13	UE	1360	1	1	0	1	1	1
14	UW	12500	1	1	0	1	1	0
15	UW	65000	1	1	0	1	1	0
16	V	7803	1	1	0	1	1	1
*Pfhrp* 2 exon 2 positive								
17	A	89	0	1	1	0	0	1
18	A	9241	1	1	1	1	1	1
19	A	26230	1	1	1	1	1	1
20	A	43944	1	1	1	1	1	0
21	BA	2495	0	1	1	1	0	1
22	BA	5665	1	1	1	1	0	1
23	C	868	0	1	1	0	0	0
24	E	37923	1	1	1	1	1	1
25	N	272	1	1	1	1	1	1
26	N	1143	1	1	1	1	0	1
27	N	2978	1	1	1	1	0	1
28	N	240	1	1	1	1	1	1
29	N	580000	1	1	1	1	1	1
30	UE	8000	1	1	1	1	1	1
31	UE	4360	1	1	1	1	1	1
32	UE	8560	1	1	1	0	1	1
33	UE	25000	1	1	1	1	1	1
34	UE	122500	1	1	1	1		1
35	UE	97500	1	1	1	1	0	1
36	UE	270000	1	1	1	1	1	0
37	UW	3262	1	1	1	1	1	1
38	UW	65000	1	1	1	1	1	1
39	UW	8465	1	1	1	1	1	1
40	UW	7000	1	1	1	1	1	1
41	UW	57500	1	1	1	1	1	1
42	UW	190000	1	1	1	1	1	1
43	UW	257500	1	1	1	1	1	1
44	UW	380000	1	1	1	1	1	1
45	V	13638	1	1	1	1	1	1
46	V	24240	1	1	1	1	1	1
47	W	139513	1	1	1	1	1	0
48	W	66049	1	1	1	1	1	1
49	W	190000	1	1	1	1	1	1

PfHRP 2, PfHRP 2 antigen; Aldo, aldolase; A, Ashanti; BA, Brong Ahafo; C, Central; E, Eastern; GA, Greater Accra; N, Northern; UE, Upper East; UW, Upper West; V, Volta; W, Western. PD, parasite density; p/μl, parasites per microliter blood. The numbers represent the number of samples that produced amplicons after the respective PCR reactions.

## Discussions

False negative PfHRP 2-based RDT results can occur as a result of compromised test quality or operator error [15]. Other causes of false negative RDT results include infections with parasite densities below the detection limit of the RDT as well as deletions of the *Pfhrp 2* gene that codes for the target antigen PfHRP 2. False negative RDT results amongst symptomatic malaria patients can lead to febrile malaria cases being treated as either bacterial or viral infections [30], which can lead to an increase in morbidity as well as a loss of patient confidence in the health system [31]. Increasing reports of the presence of *Pfhrp* 2 gene deletions in parasites circulating in malaria endemic countries including Ghana [19, 20, 32–34] lead to this systematic study in which the WHO recommended surveillance protocol was used to estimate the prevalence of *Pfhrp* 2 and *Pfhrp* 3 gene deletions in parasites from febrile malaria patients attending selected health facilities across all the Regions of Ghana.

The PfHRP 2 based malaria RDTs presently in use in Ghana remains very effective as the prevalence of false negative RDT results identified in this study was less than 5%, out of which about 10% were contained in infections with parasite densities below 200 p/μl and likely below the detection threshold of the RDT. Although false negative RDT results were identified in all the 10 Regions, they were lower in two neighboring Regions in the South-Western (Western and Eastern Region) and the South Eastern (Volta and Greater Accra) parts of the country. The low number of false negative RDT results identified in this study is lower than 8.4% and 54% that have been reported in Uganda and Benin respectively [35]. Low parasite density infections are known to causes false negative RDT results [35] as has been reported from India and the Amazon basin [12, 36].

This study identified parasites with *Pfhrp* 2/3 gene deletions in samples from symptomatic malaria patients. Parasites with *Pfhrp* 2 exon 2 gene deletions accounted for about a third of the false negative samples. Parasites with *Pfhrp* 2/3 gene deletions were identified in all but one of the Regions in Ghana, the Eastern Region where no sample was identified to contain parasites with deletions in exon 2 of the *Pfhrp* 2 gene. All parasites with deletions in *Pfhrp* 2 exon1-2 also had deletions in *Pfhrp* 2 exon 2 and were mainly identified in 3 neighboring Regions in the lower mid-section of western Ghana. The pattern of distribution of parasites with deletions in both exon 2 and exon 1–2 of the *Pfhrp* 3 gene were like that of the *Pfhrp* 2 gene. Deletions at exon 2 were three times more prevalent than at exon 1–2 for both the *Pfhrp* 2 and *Pfhrp* 3 genes. The observed higher prevalence of deletions in exon 2 than exon 1–2 could be a true reflection of parasites with exon 2 gene deletions being more abundant than parasites with exon 1–2 gene deletions as has been reported in other studies such as an earlier report from India where parasites with *Pfhrp* 2 exon 2 gene deletions were at higher frequencies than parasites with *Pfhrp* 2 exon 1–2 gene deletions [12]. The variations in the number of amplicons obtained after the exon 1–2 and the exon 2 PCR amplifications could however be an artifact of the PCR methods used in amplifying the two fragments, as the nested PCR used for exon 1–2 PCR is more sensitive than the one step PCR used for exon 2 genotyping. Other possible reasons for the variation in the number of amplicons obtained after exon 2 and exon 1–2 PCR is the possibility of polymorphisms in the 3’ end(s) of the primer binding sites of exon 2 which are absent in the exon 1–2 region as well as partial degradation of the DNA template as has been previously suggested [37, 38]. Despite the possibility of false negative *Pfhrp* 2 exon 2 PCR results, some studies have identified samples with higher frequencies of deletions in *Pfhrp* 2 exon 1–2 than *Pfhrp* 2 exon 2 [39].

Parasites with intact *Pfhrp* 3 genes have been found to compensate for the absence of *Pfhrp* 2 exon 2 in a parasite, especially at high parasite densities and result in a positive PfHRP 2 based malaria RDT test due to the cross-reactivity of PfHRP 3 antigen with the PfHRP 2 antibodies on the RDT kits [9, 40]. However, the presence of parasites with the *Pfhrp* 3 exon 2 gene and most likely the corresponding PfHRP 3 antigen in circulation in the false negative samples identified in this study did not result in a positive PfHRP 2 RDT result, even though some of the samples had parasite densities of greater than 1000 parasites per microlitre. The absence of PfHRP 3 antigen compensation has been observed in other studies including one from Kenya [40] and Nigeria [32]. PfHRP 3 antigen cross-reactivity with PfHRP 2 antibodies (PfHRP 3 antigen compensation) could have occurred in some of the RDT positive samples, however; this was not explored in this study.

Despite the high positive correlation between parasite density and aldolase activity, about 38% of the false negative samples had aldolase levels below the cut off for positivity, which suggests that some parasites isolates produced very low aldolase activity. The presence of PfHRP 2 antigen in the false negative samples that had no aldolase activity could have resulted from persisting PfHRP 2 antigen from a recently cleared infection containing parasites with intact *Pfhrp* 2 exon 2 or due to the presence of some parasite isolates producing very low concentrations of aldolase. The prozone effect could have been one of the reasons why samples containing high concentration of PfHRP 2 antigen tested negative by the PfHRP 2 based RDT kit as has been suggested in an earlier report [41].

There were about a third of the aldolase positive false negative RDT results that were caused by parasites with *Pfhrp* 2 exon 2 gene deletions. The confirmation of parasites with *Pfhrp* 2 gene deletions amongst symptomatic malaria patients with false negative RDT results is detrimental to malaria control in Ghana, especially in communities and facilities where malaria diagnosis is solely based on RDT results. The prevalence of parasites with deletions in the *Pfhrp* 2 gene circulating in symptomatic malaria patients could be more than identified in this study as only parasites contained in false negative samples were evaluated as opposed to all microscopy positive samples. Overall, the low prevalence of samples classified as false negative resulted in only a small fraction of the samples collected analyzed for *Pfhrp* 2 gene deletions.

## Conclusion

The low prevalence but nationwide distribution of samples with false negative RDT test results provides assurance that PfHRP 2 based malaria RDT kits remain effective in diagnosing symptomatic malaria across all the Regions of Ghana. Although there was a low prevalence of parasites with deletions in both exon 2 and exon 1–2 of the *Pfhrp* 2 gene the prevalence of parasites with deletions in *Pfhrp* 2 exon 2 was about a third of the false negative RDT results. The need to ensure rapid, accurate and reliable malaria diagnosis requires continuous surveillance and monitoring of parasites with *Pfhrp* 2 gene deletions.

## Supporting information

S1 Table Primer details. (DOCX)

S2 Table Properties of the 127 samples used in the study. (XLSX)

S3 Table Properties of the 31 samples with *Pfhrp* 2 exon 2 gene deletions. (XLSX)
