# Supplementary material for: Contribution of P. falciparum parasites with Pfhrp 2 gene deletions to false negative PfHRP 2 based malaria RDT results in Ghana: A nationwide study of symptomatic malaria patients
Source: PLoS One. 2020 Sep 4;15(9):e0238749. doi: 10.1371/journal.pone.0238749 (PMC7473533; doi:10.1371/journal.pone.0238749)
Supplement: S1 Table — (DOCX) [file pone.0238749.s001.docx]

S1 Table. Primer details. Information on all the primers used in the study

| PCR | Primer | Primer Sequence (5’-3’) |
| --- | --- | --- |
| *Plasmodium* spp |  |  |
| Primary | rPLU6 | TTAAAATTGTTGCAGTTAAAA CG |
|  | rPLU5 | CCTGTTGTTGCCTTAAACTTC |
| *Secondary* |  |  |
| *P. falciparum* | rFAl1 | TTAAACTGGTTTGGGAAAACCAAATATATT |
|  | rFAL2 | ACACAATGAACTCAATCATGACTACCCGTC |
| *Msp* |  |  |
| Primary | M1-OF | CTAGAAGCTTTAGAAGATGCAGTATTG |
|  | M1-OR | CTTAAATAGTATTCTAATTCAAGTGGATCA |
| Secondary |  |  |
| K1 | M1-KF | AAATGAAGAAGAAATTACTACAAAAGGTGC |
|  | M1-KR | GCTTGCATCAGCTGGAGGGCTTGCACCAGA |
| RO33 | RO33-RF | TAAAGGATGGAGCAAATACTCAAGTTGTTG |
|  | RO33-R2 | CAAGTAATTTTGAACTCTATGTTTTAAATCAGCGCGTA |
| Mad20 | M1-MF | AAATGAAGGAACAAGTGGAACAGCTGTTAC |
|  | M1-MR | ATCTGAAGGATTTGTACGTCTTGAATTACC |
| *Glurp* |  |  |
| Primary | GF3 | ACATGCAAGTGTGATCCTGAA |
|  | GF4 | TGTAGGTACCACGGGTTCTTGTGG |
| Secondary | GF4 | TGTAGGTACCACGGGTTCTTGTGG |
|  | GNF | TGTTCACACTGAACAATTAGATTTAGATCA |
| *Pfhrp* 2 Exon 1-2 |  |  |
| Primary | Hrp2.1 | GGTTTCCTTCTCAAAAAATAAAG |
|  | Hrp2.2 | CGAAACTCAAGCACATGTAGA |
| Secondary | Hrp2.3 | GTATTATCCGCTGCCGTTTTTGCC |
|  | Hrp2.4 | TTCCGCATTTAATAATAACTTGTGTAG |
| *Pfhrp* 3 Exon 1-2 |  |  |
| Primary | Hrp3.1 | GGTTTCCTTCTCAAAAAATAAAA |
|  | Hrp3.2 | CCTGCATGTGCTTGACTTTA |
| Secondary | Hrp3.3 | ATATTATCGCTGCCGTTTTTGCT |
|  | Hhrp3.4 | CTAAACAAGTTATTGTTAAATTCGGAG |
| *Pfhrp* 2 Exon 2 | Pfhrp2F1 | CAAAAGGACTTAATTTAAATAAGAG |
|  | Pfhrp2R1 | AATAAATTTAATGGCGTAGGCA |
| *Pfhrp* 3 Exon 2 | Pfhrp3F1 | ATGCAAAAGGACTTAATTC |
|  | Pfhrp3R1 | TGGTGTAAGTGATGCGTAGT |
